# Effects of Litter on Seedling Emergence and Seed Persistence of Three Common Species on the Loess Plateau in Northwestern China

**DOI:** 10.3389/fpls.2017.00103

**Published:** 2017-01-31

**Authors:** Rui Zhang, Xiaowen Hu, Jerry M. Baskin, Carol C. Baskin, Yanrong Wang

**Affiliations:** ^1^State Key Laboratory of Grassland Agro-Ecosystems, College of Pastoral Agriculture Science and Technology, Lanzhou UniversityLanzhou, China; ^2^Department of Biology, University of Kentucky, LexingtonKY, USA; ^3^Department of Plant and Soil Sciences, University of Kentucky, LexingtonKY, USA

**Keywords:** grassland ecosystem health, *Lespedeza davurica*, litter, Loess Plateau, seedling emergence, seed persistence, *Setaria glauca*, *Stipa bungeana*

## Abstract

Litter accumulation resulting from land use change (enclosure) is one of the key variables influencing seedling recruitment and consequently the regeneration of plant populations and seed persistence in the soil seed bank. A better understanding of the effects of litter on seed germination and seedling emergence is crucial for developing a new set of indicators for grassland ecosystem health and for grassland management policy. We investigated the effects of seed position in litter and amount of litter covering the seed on seedling emergence and seed persistence of three common species on the Loess Plateau in northwestern China. Seed position beneath the litter layer provided a suitable environment for seedling emergence of the three species. A moderate amount of litter (160 g/m^2^) was beneficial for seedling emergence of the small-seeded species *Stipa bungeana* and *Lespedeza davurica* from seeds from beneath the litter layer. The large-seeded species *Setaria glauca* was more tolerant of a high amount of litter (240 g/m^2^) than the two small-seeded species. Seed persistence in the soil differed among the three species and also was affected by seed position in litter and amount of litter cover. The proportion of viable seeds of *Stipa bungeana* and *Setaria glauca* on top of the litter layer increased with an increase in amount of litter. Seedling emergence and seed persistence varied significantly among species, amount of litter and seed position in litter. A moderate amount of litter and seeds positioned beneath the litter layer were better for seedling recruitment than for those on top of the litter layer. A high amount of litter was more favorable for persistence of seeds positioned on top of the litter than for those beneath the litter. Our study showed that maintaining litter amount between 80 and 160 g/m^2^ is optimal for *S. bungeana* dominated grassland on the Loess Plateau. We suggest that litter amount can serve as a guide for monitoring and managing grassland ecosystems, as it is an indicator of ecosystem processes that are essential for biodiversity conservation and restoration.

## Introduction

Litter plays an important role in influencing community structure (Grime, 2001) and may affect the establishment of new individuals as a form of post-death, plant–plant interaction (Facelli and Pickett, 1991a). A litter layer acts as a mechanical barrier to seedling emergence, changes physical factors of the environment, such as soil temperature and moisture and light quantity and quality and indirectly modifies the chemical environment by releasing allelochemicals that affect seedling establishment and seedling growth (Facelli and Pickett, 1991a; Holmgren et al., 1997; Ruprecht et al., 2010a). Thus, litter may influence the diversity and dynamics of plant communities, and consequently productivity and health of ecosystem (Fowler, 1986; Gibson and Good, 1987; Facelli and Pickett, 1991b; Quested et al., 2005; Ruprecht et al., 2010b; Eckstein et al., 2011).

The effect of litter on seedling emergence and consequently seedling recruitment varies with environmental conditions, litter amount, seed position and seed traits (Hovstad and Ohlson, 2008; Wellstein, 2012; Egawa and Tsuyuzaki, 2013; Loydi et al., 2013). A meta-analysis showed that litter had an overall negative effect on seed germination and seedling establishment in grassland, old field and forest ecosystems (Xiong and Nilsson, 1999), but recent studies indicated that this might not be the case in grassland ecosystems (Quested and Eriksson, 2006; Donath and Eckstein, 2008; Loydi et al., 2013). For dry grasslands or grasslands under water-limited conditions, low to medium amounts of litter had a positive effect on seedling emergence and recruitment. However, a high amount of litter will inhibit seed germination and seedling establishment by reducing light quality and quantity beneath the litter layer, by preventing seeds on top of the litter from reaching the soil or by preventing roots of seedlings that germinate on top of the litter layer from reaching the soil surface (Xiong and Nilsson, 1999; Eckstein and Donath, 2005; Donath and Eckstein, 2010; Loydi et al., 2013).

In addition to amount of litter, the responses of plants to litter depend on seed position in/on the litter layer. Seeds can be positioned on top, within or beneath the litter layer depending on whether litter input occurs before, during or after seed rain (Wellstein, 2012). For example, seedling emergence of *Pimpinella saxifraga, Leontodon autumnalis*, and *Sanguisorba officinalis* was significantly higher for seeds beneath the litter layer than for those on top of it or on the bare soil (Wellstein, 2012). In contrast, germination of forest understory *Carex* species was lower for seeds beneath the leaf litter than for those on top of the litter layer (Vellend et al., 2000).

The soil seed bank is an important consideration in community ecology, and above-ground vegetation dynamics, and knowledge of seed persistence in the soil plays an important role in the conservation of rare species, in maintenance of plant communities and in restoration of vegetation following disturbances (Van der Valk and Verhoeven, 1988; Hodgson and Grime, 1990; Jones and Esler, 2004; Bossuyt and Honnay, 2008; Michaela and Wolfgang, 2009; Baskin and Baskin, 2014; Long et al., 2015). Seed persistence is affected by many factors, such as seed characteristics, species characteristics and abiotic and biotic conditions in the pre-dispersal and post-dispersal environments (Long et al., 2015). Kettenring et al. (2006) reported that seed bank development is not only determined by the standing vegetation but also by modification of microenvironments through litter accumulation. Litter derived from the standing vegetation can act as a seed trap and thus prevent seed germination. Non-germinated seeds in the soil are the prime determinant of the composition of the seed bank, and development of a thick layer of litter may promote seed bank development (Egawa et al., 2009).

The Loess Plateau in China is characterized by an arid to semiarid climate. Heavy rain storms easily erode the loess soil, and low vegetation cover and degradation of grasslands by overgrazing have long been a serious problem that has hugely impacted grassland animal husbandry and ecological security in this area (Unkovich and Nan, 2008; Zhao et al., 2013). To prevent further soil erosion and desertification and to protect and restore the grassland, the Chinese government implemented the Returning Farmland to Grassland and Returning Rangeland to Grassland programs in 2000 and 2003, respectively (Lin et al., 2013). However, the effects of restoration programs on grasslands are controversial. Some studies showed that ecological restoration programs had a positive effect on vegetation (Pettit et al., 1995; Liu et al., 2008), and others indicated that reasonable grazing or mowing was conducive to maintenance of the natural vegetation (Dumont et al., 2009; Cheng et al., 2014). Further, long-term enclosure had a negative effect on plant regeneration in semiarid areas on the Loess Plateau due to the accumulation of litter that inhibited seed germination and thus indirectly affected natural regeneration (Cheng et al., 2006). Thus, litter accumulation during ecological restoration may play a key role in the productivity and health of the grassland ecosystem on the Loess Plateau. Understanding the role of litter in regulating seed germination, seedling recruitment and the soil seed bank will aide in developing a new set of indicators for grassland ecosystem health.

Yet, there is little information (Rotundo and Aguiar, 2005; Liu et al., 2016) on how amount of litter and seed position in the litter jointly influence seedling emergence and seed persistence; specifically, no such study has been conducted on the Loess Plateau. *Stipa bungeana*, a dominant perennial grass on the Loess Plateau, plays an important role in protecting the soil from erosion and reducing water loss by runoff (Hu et al., 2014). *Setaria glauca*, a summer annual grass, is native to Eurasia (Steel et al., 1983), but it has become a cosmopolitan grass weed throughout the temperate region (Culpepper and Sheldon, 1999) and is common in the study area. *Lespedeza davurica*, a C_3_ perennial leguminous shrub, is a dominant species in the natural grassland community on the Loess Plateau of China (Xu et al., 2013). Also the dominant species *S. bungeana* produces a relatively high amount of litter during the growing season. Thus, we select these three common species and the litter of *S. bungeana* as object to answer the following questions. (1) What amount of litter is beneficial for seedling emergence and seed persistence of the dominant species *Stipa bungeana* and its accompanying species *Setaria glauca* and *Lespedeza davurica*? (2) How do seed position and its interaction with amount of litter affect seedling emergence and seed persistence?

## Materials and Methods

### Study Site and Species

Field experiments were conducted from 28 July 2013 to 28 December 2014 on Yuzhong Campus (N35°57′, E104°09′, 1720 m above sea level) of Lanzhou University, Gansu Province, China. Precipitation and temperature data collected on the Yuzhong Campus showed that mean annual precipitation is 350 mm, 60% of which occurs from July to September, and mean annual temperature is 6.7°C.

### Seed Collection

Seeds of *S. bungeana* and *S. glauca* were collected at the Yuzhong experimental station in July 2013 and those of *L. davurica* at the same site in October 2012. Seeds of each of the three species were collected from more than 50 individuals and kept in paper bags at room temperature (20–45% RH; 18–25°C) until used in experiments. Other than the after-ripening that may have occurred during storage, no dormancy-breaking treatments were given to the seeds before they were used in the experiments. The 1000-seed mass determined using eight replicates of 100 seed samples was 1.14 ± 0.04 g, 3.56 ± 0.05 g and 1.86 ± 0.04 g for *S. bungeana, S. glauca*, and *L. davurica*, respectively.

### Seedling Emergence: Effect of Seed Position in Litter, Species and Amount of Litter

The effects of seed position in litter, species and amount of litter on seedling emergence were studied in the field on the Yuzhong Campus from 28 July to 6 October 2013, during the rainy season. Cheng et al. (2006) indicated that the amount of litter was 40–90, 80 –140, 120–280, and 160–240 g/m^2^ in *Stipa bungeana*-dominated grassland enclosed for 0–5, 6–10, 11–15, and 16–20 years, respectively, and that it peaked 267 g/m^2^ for this grassland enclosed for 11–15 years. Thus, we used 0, 80, 160, and 240 g/m^2^ to mimic the natural conditions. There were seven treatments: control (no litter, seeds placed on bare soil), seeds on top of 80 g/m^2^ litter layer, seeds beneath the 80 g/m^2^ litter layer, seeds on top of 160 g/m^2^ litter layer, seeds beneath 160 g/m^2^ litter layer, seeds on top of 240 g/m^2^ litter layer and seeds beneath 240 g/m^2^ litter layer. Thus, there were three seed positions (on top of the litter layer, beneath litter layer, on bare soil = no litter) and four amounts of litter (0, 80, 160, 240 g/m^2^). One hundred and five 20-cm long × 18 cm diameter PVC collars (pots) (about 5.0 × 10^3^ cm^3^) were used, and each treatment was replicated 15 times (7 treatments × 15 replications = 105 pots). Pots were filled with soil from the natural habitat that had been screened through a 40-mesh screen to remove any seeds of the three study species present in the seed bank. Fifty seeds of each of the three species were sown in 105 pots (150 seeds per pot) on 28 July 2013. Any seeds of *S. bungeana* in the litter were removed before the litter was used. After seeds and litter were added to the pots, they were covered with 15 cm × 15 cm 40-meshscreensto prevent unintentional seed input and seed predation. The bottom of each pot was covered on the outside with nylon mesh to prevent loss of seeds through the drainage holes. The number of seedlings that emerged was recorded each week for 10 weeks, and seedlings were removed from the pots after they were counted. Seedlings of the two grasses were distinguished based on width of the leaf: leaves of *Stipa* were narrow like needles and those of *Setaria* were significantly wider than those of *Stipa*.

### Seed Persistence: Effect of Seed Position in Litter and Amount of Litter

This experiment began on 28 July 2013 and ended 28 December 2014. The experimental design was the same as that described for seedling emergence. Seeds were retrieved from the pots on 28 December 2013, 28 June 2014 and 28 December 2014. For each species, seven treatments with five replicates each, i.e., 35 pots, were retrieved on each of the three dates, and the soil within each pot was sieved through a 40-mesh screen. Then, the number of seeds of each species remaining was counted and tested for viability via germination tests and the embryo cut test. Seeds with white and firm embryos were considered to be viable and those with soft and tan embryos non-viable. Dead non-germinated and dead germinated seeds could not be distinguished from each other.

### Statistical Analysis

A three-way ANOVA was used to test the effect of seed position in litter, species and amount of litter on seedling emergence and effect of seed position in the litter, duration in the field (time since sowing) and amount of litter on seed persistence in soil. Percentages of viable seeds were log transformed before analysis, but only non-transformed data are shown in tables and figures. Duncan’s multiple range tests were used to compare means among treatments. Data were analyzed using SPSS 21.0 software and the figures were created with Excel 2007.

## Results

### Seedling Emergence: Effect of Seed Position in Litter, Species and Amount of Litter

**FIGURE 1:**
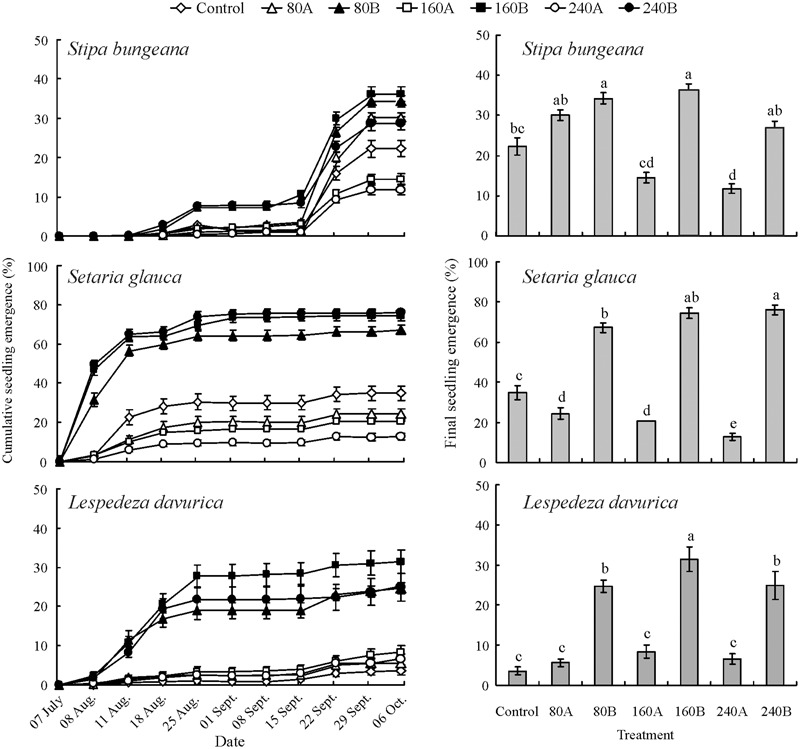
**Effect of litter on cumulative seedling emergence (mean% ± SE) of *Stipa bungeana, Setaria glauca*, and *Lespedeza davurica*.** Bars with different lowercase letters differ significantly among different treatments within the same species. Zero (control), 80, 160, and 240 refer to amount of litter (g/m^2^), and A and B to seed positioned on top of (A) or beneath (B) the litter layer.

Seed position in litter, species, amount of litter and their interactions had significant effects on cumulative seedling emergence. In general, seedling emergence of the three study species differed significantly (**Table 1**, *P* < 0.001). Seedling emergence of *S. bungeana* and *S. glauca* decreased with an increase in amount of litter for seeds sown on top of litter. However, seedling emergence of *L. davurica* did not differ significantly among different amounts of litter for seeds on top of the litter layer. For seeds sown beneath the litter layer, cumulative seedling emergence of each of the three species was significantly higher than that for seeds sown on top of the litter layer or directly on the bare soil (control = no litter) (**Figure 1**).

**Table 1:** Effect of seed position (P), species (S), amount of litter (L) and their interactions on cumulative seedling emergence.

Source of variation	*df*	Mean square	*F*	*P*
Position (*P*)	1	10692.900	374.413	<0.001
Species (*S*)	2	6671.233	233.594	<0.001
Amount of litter (*L*)	3	578.885	20.270	<0.001
*P* × *S*	2	1922.533	67.318	<0.001
*P* × *L*	3	1327.152	46.470	<0.001
*S* × *L*	6	127.919	4.479	<0.001
*P* × *S* × *L*	6	261.930	9.171	<0.001

The highest cumulative seedling emergence of *S. bungeana, S. glauca*, and *L. davurica* was 36.13 ± 3.64%, 76.00 ± 2.25% and 31.47 ± 3.04%, respectively, for seeds sown beneath 160, 240, and 160 g/m^2^ of litter, respectively. For *L. davurica*, seed position in the litter had a significant effect on cumulative seedling emergence, which was lower for seeds on top of the litter layer and on bare ground than for those positioned beneath the litter layer (**Figure 1**).

### Seed Persistence: Effect of Seed Position in Litter and Amount of Litter

**FIGURE 2:**
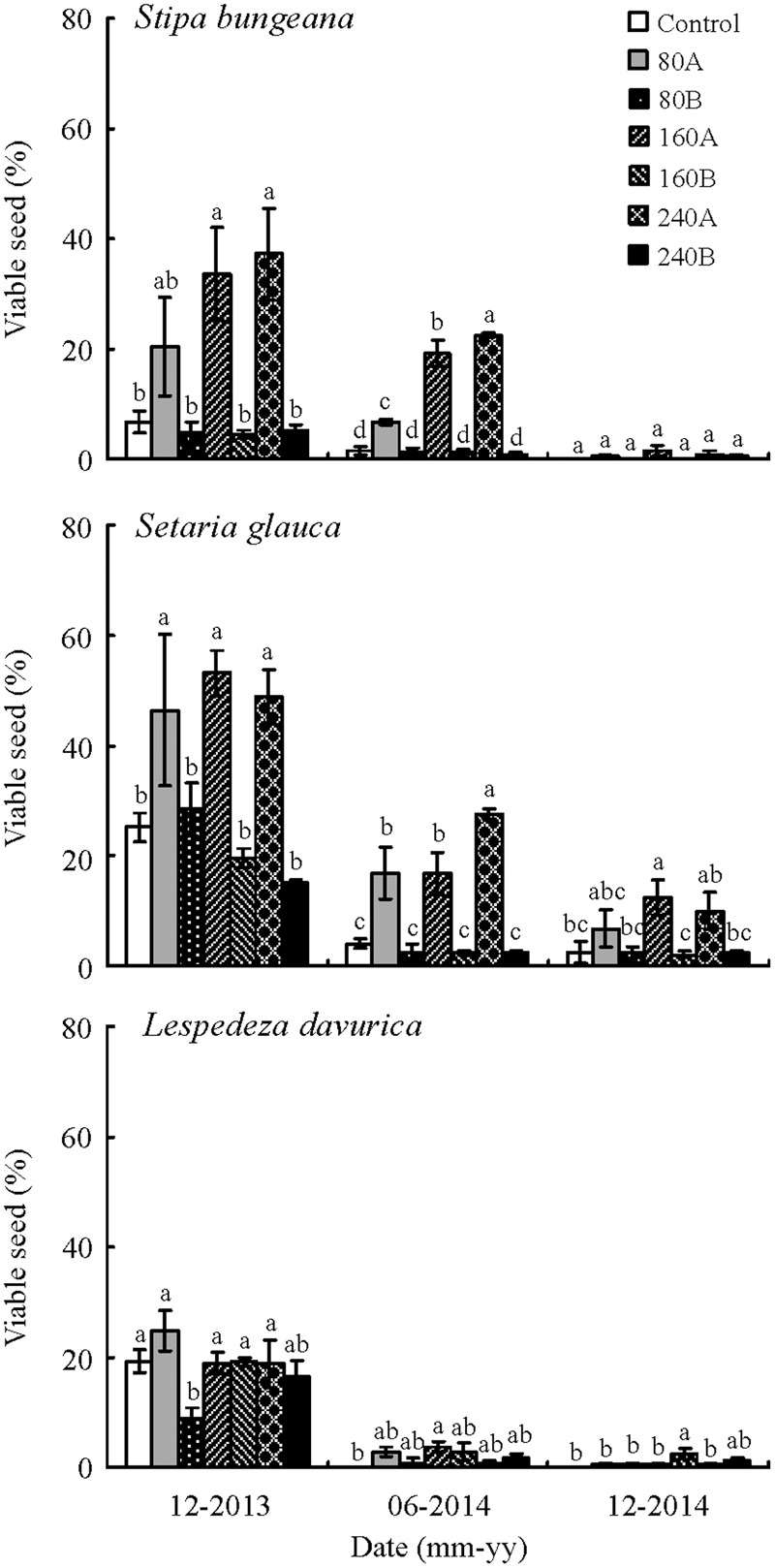
**Effect of seed position and amount of litter on seed viability (mean% ± SE) of *Stipa bungeana, Setaria glauca*, and *Lespedeza davurica* in the field under the seven treatments on three dates.** Bars with different lowercase letters differ significantly among different treatments within the same sample date. Zero (control), 80, 160, and 240 refer to amount of litter (g/m^2^), and A and B to seed positioned on top of (A) or beneath (B) the litter layer.

For all three species, percentage of viable seeds in the soil decreased significantly with an increase of time (duration) in the field (**Figure 2**). Seed position, seed duration in the field (time since sowing), amount of litter and some of their interactions (duration × amount of litter and position × duration × amount of litter) had significant effects on seed persistence of *S. bungeana* (**Table 2**) and *S. glauca* (**Table 3**). However, seed position and seed position × duration × amount of litter did not have a significant effect on seed persistence of *L. davurica* (**Table 4**).

**Table 2:** Three-way ANOVA of the effects of seed position (P), duration (D), amount of litter (L), and their interaction on seed viability of *Stipa bungeana* in the field.

Source of variation	*df*	Mean square	*F*	*P*
Position (*P*)	1	6.220	83.075	<0.001
Duration (*D*)	2	7.316	97.712	<0.001
Litter amount (*L*)	3	0.664	8.870	<0.001
*P* × *D*	2	1.105	14.756	<0.001
*P* × *L*	3	0.847	11.306	<0.001
*D* × *L*	6	0.091	1.218	0.304
*P* × *D* × *L*	6	0.158	2.114	0.058

**Table 3:** Three-way ANOVA of the effects of seed position (P), duration (D), amount of litter (L), and their interaction on seed viability of *Setaria glauca* in the field.

Source of variation	*df*	Mean square	*F*	*P*
Position (*P*)	1	4.279	42.054	<0.001
Duration (*D*)	2	8.950	87.963	<0.001
Litter amount (*L*)	3	0.581	5.708	0.001
*P* × *D*	2	0.647	6.363	0.003
*P* × *L*	3	0.520	5.113	0.003
*D* × *L*	6	0.157	1.542	0.173
*P* × *D* × *L*	6	0.100	0.985	0.440

**Table 4:** Three-way ANOVA of the effects of seed position (P), duration (D), amount of litter (L), and their interaction on seed viability of *Lespedeza davurica* in the field.

Source of variation	*df*	Mean square	*F*	*P*
Position (*P*)	1	0.010	0.237	0.627
Duration (*D*)	2	14.817	349.016	<0.001
Litter amount (*L*)	3	0.255	6.010	0.001
*P* × *D*	2	0.214	5.029	0.008
*P* × *L*	3	0.207	4.867	0.003
*D* × *L*	6	0.117	2.754	0.016
*P* × *D* × *L*	6	0.044	1.027	0.413

Percentage of viable seeds in the field varied with seed position in litter, seed duration in the field and amount of litter. Percentage of viable seeds of *S. bungeana* was significantly higher for seeds on top of the litter layer than it was for those positioned beneath the litter layer or on bare ground, and it increased with an increase in amount of litter. After 5 months, 37.2% of *S. bungeana* seeds positioned on top of 240 g/m^2^ litter were viable, whereas only 5.2% of those positioned beneath the same amount of litter were viable (**Figure 2**). Percentage of viable seeds of *S. glauca* in the field was significantly affected by seed position. After 5 months in the field, 46.4–53.2% of seeds positioned on top of the litter layer were viable, but only 15.2–28.4% of those positioned beneath different amounts of litter were viable. Decrease in number of viable seeds of *S. glauca* in the field was slower with an increase in time of burial compared to the other two species, and 2.4–12.4% seeds were still viable after 1.5 year under different treatments (**Figure 2**). In contrast to *S. glauca* and *S. bungeana*, seed position of *L. davurica* did not have a significant effect on seed persistence. Percentage of viable seeds of *L. davurica* decreased quickly and was ≤3.6% for all the treatments after duration of 11 months in the field (**Figure 2**).

## Discussion

### Seedling Emergence: Effect of Seed Position in Litter, Species and Amount of Litter

The effect of litter on seed germination and seedling establishment in grasslands ranged from strongly negative to slightly positive depending on environmental conditions, amount of litter and seed traits (Loydi et al., 2013). Our study showed that the effect of littler on seedling emergence ranged from slightly positive (*S. bungeana*) to positive (*S. glauca* and *L. davurica*) for seeds beneath the litter layer compared to those on bare soil. For seeds positioned on top of the litter layer, seedling emergence was slightly promoted, unchanged or inhibited depending on species and amount of litter compared to those on bare ground. Seed position in litter, species (seed traits) and amount of litter had significant effects on seedling emergence of the three common species on the Loess Plateau.

Low to medium amounts of litter (<500 g/m^2^) had a positive effect on seedling recruitment in dry grasslands or under water-limited conditions (Loydi et al., 2013). This is consistent with our study, which clearly showed that litter had a positive effect on seedling emergence of all three study species when seeds were beneath the litter layer compared to those on bare soil. However, the beneficial effect for seeds beneath the litter layer varied with litter amount and species. For example, seedling emergence increased and then decreased as amount of litter increased in *S. bungeana* and *L. davurica*. However, a continual increase in seedling emergence with an increase in litter amount in *S. glauca* was observed. A possible interpretation of this result is that the presence of litter may maintain soil moisture or reduce the intensity of desiccation of seeds, which would facilitate germination and seedling establishment in a dry environment or during drought (Boeken and Orenstein, 2001; Eckstein and Donath, 2005; Rotundo and Aguiar, 2005; Donath and Eckstein, 2010; Loydi et al., 2013). Further, seedlings from seeds beneath the litter layer must reach light levels sufficient for photosynthesis, and a thick litter layer may reduce seedling emergence due to the lack of light (Facelli and Pickett, 1991b; Wellstein, 2012). Compared with small-seeded species, large-seeded species such as *S. glauca* can better cope with a thick litter layer (Krenova and Leps, 1996; Loydi et al., 2013), since they have sufficient resources for elongation of the hypocotyl to penetrate the litter layer and thus reach full sunlight (Hamrick and Lee, 1987).

However, when seeds were placed on top of the litter layer, seedling emergence was promoted, unchanged or inhibited depending on species and amount of litter. Seedlings from seeds on top of the litter layer must quickly make contact with the soil to order to avoid lethal desiccation (Hamrick and Lee, 1987). Seedling emergence of *S. bungeana* for seeds on top of 80 g/m^2^ litter layer was slightly higher more than that of seeds sown on bare soil, indicating that resource investment for elongation of the radical allows *S. bungeana* to cope with this low amount of litter cover. For *L. davurica*, seedling emergence for seeds on top of the litter layer did not differ significantly from those on bare soil. This showed that the positive effects of litter, such as attenuating temperature extremes and reducing water stress (Eckstein and Donath, 2005; Donath and Eckstein, 2010), was equal to the negative effect of litter, such as reducing soil-seed contact (Fowler, 1986; Chambers, 2000; Wellstein, 2012). However, the negative effects of litter outweighed its possible positive effects for *S. glauca*, and seedling emergence was significantly inhibited for seeds on top of the litter layer. Our findings highlight that the amount of litter in grassland ecosystems may serve as a potential indicator of plant diversity and composition over time due to its effects on timing of seed dispersal and seedling emergence.

### Seed Persistence: Effect of Seed Position in Litter and Amount of Litter

Litter can modify soil temperature and soil moisture (Mackinney, 1929; Fowler, 1986; Boeken and Orenstein, 2001), and previous studies indicated that seed persistence can be affected by soil temperature, water potential and light transmission through their influence on germination, dormancy and aging (Long et al., 2009, 2015; Pakeman et al., 2012). Litter increased seed longevity of *Bromus pictus* through ameliorating temperature, which is one of the principal drivers of seed aging (Rotundo and Aguiar, 2005).

The presence of a litter layer may maintain soil moisture (Fowler, 1986; Boeken and Orenstein, 2001) and reduce temperature (Chambers and MacMahon, 1994), and temperature and moisture content are important factors that influence seed aging (Walters, 1998). Compared to seed position on top of the litter layer, its position beneath the litter layer had higher moisture content and lower temperature, and the moisture content of a seed at a given relative humidity increases as the temperature decreases (Roberts and Ellis, 1989; Facelli and Pickett, 1991b; Walters, 1998). Lower soil temperature and water availability promote persistence of desiccation-tolerant seeds (Long et al., 2009, 2015; Pakeman et al., 2012) by providing conditions that minimize seed aging, dormancy loss (Davis et al., 2005) and microbial activity (Schafer and Kotanen, 2003). Thus, the effect of litter on seed persistence depends on whether temperature or soil moisture has a greater effect on seed longevity. Our study showed that on the Loess Plateau seeds of *S. bungeana* and *S. glauca* on top of the litter layer persist longer than those on the bare soil or beneath the litter layer, which indicates that soil moisture plays a more important role in seed persistence than temperature.

Percentage of viable seeds of *S. bungeana* was 20.4, 33.6, and 37.2% for seeds positioned on top of 80, 160, and 240 g/m^2^ litter layer, respectively, after 5 months, whereas only 6.8% of those on bare soil were viable. After 11 months, percentage of viable seeds was 6.8, 19.2, and 22.4% for seeds positioned on top of 80, 160, and 240 g/m^2^ litter layer, respectively, all of which were significantly higher than those beneath different amounts of litter layer and on bare soil (1.6%). Hu et al. (2014) reported that all *S. bungeana* seeds had lost viability after 5 months of burial at 5 cm, whereas 12 and 4% of those on the soil surface were viable after 5 and 12 months, respectively. Our results indicated that litter had a positive effect on seed persistence, and percentage of viable seeds increased with amount of litter for seeds on top of litter layer. One possible interpretation of this result is that seeds of *S. bungeana* have a long awn that causes them to be easily trapped in the litter, thus preventing seed contact with the surrounding soil. As such, the seeds failed to germinate or germination was delayed (Ruprecht and Szabo, 2012).

Seeds of *S. glauca* have been reported to live for 15 (Dawson and Bruns, 1975), 30 (Kivilaan and Bandurski, 1981) and 38 (Toole and Brown, 1946) years in soil. Thus, they can form a long-lived persistent seed bank. However, in our study seeds of *S. glauca* lost viability after 11 months. Seedling emergence of *S. glauca* from August to October was >67% for seed beneath the litter layer with different amounts of litter, and it was 12.7% even for those on top of 240 g/m^2^ litter layer. As time increases, most *S. glauca* seeds could germinate if exposed to light and adequate soil moisture. Seeds of this species buried under natural temperatures in Kentucky (USA) in November and exhumed in June and July germinated to 70–100% in light and darkness at 15/6, 20/10, 25/15, 30/15, and 35/20°C (Baskin et al., 1996).

Seed persistence of *S. glauca* and *S. bungeana* was affected by amount of litter and seed position in the litter layer. However, seed persistence of *L. davurica* was affected by amount of litter but not by position in the litter. The large seeds of *S. glauca* and the awned seeds of *S. bungeana* are suspended within the litter or lodged on top of it and thus do not make contact with soil. However, the small seeds of *L. davurica* on top of the litter layer may ‘percolate’ through the litter layer and then germinate when conditions are favorable for them to do so (Rotundo and Aguiar, 2005; Ruprecht and Szabo, 2012). The fact that the soil seed bank drives multiple ecosystem functions related to community dynamics and composition suggests that seed persistence should be taken into account in assessing the impact of changes in land use and climate.

## Practical Implications

The present study clearly demonstrates that litter plays a key role in regulating seed germination, seedling emergence and seed persistence. Specifically, a moderate litter amount favors seedling emergence, whereas continual increasing litter decrease seedling emergence. These results imply that moderate utilization will be beneficial for vegetation restoration of long-term enclosure grassland, in which the amount of litter is high. Also, we propose that the amount of litter could be a good indicator for effective restoration and for grassland management. Our study showed that maintaining the amount of litter between 80–160 g/m^2^ is optimal for *S. bungeana*-dominated grassland on the Loess Plateau. Moreover, we also found that seeds beneath litter significantly improved seedling emergence regardless of litter amount, suggesting that moderate disturbance favoring downward movement of seeds may accelerate vegetation restoration. Our study supports the notion that litter accumulation resulting from long term enclosure decreases the capability of grassland regeneration and suggests that moderate utilization is necessary for maintaining a healthy grassland ecosystem on the Loess Plateau, with litter as a potential indicator.

## Author Contributions

RZ, XH, and YW conceived the topic. RZ performed the experiments. RZ and XH analyzed all statistical data. RZ and XH wrote the manuscript. JB and CB revised several version of the manuscript.

## Conflict of Interest Statement

The authors declare that the research was conducted in the absence of any commercial or financial relationships that could be construed as a potential conflict of interest. The reviewer NK and handling Editor declared their shared affiliation, and the handling Editor states that the process nevertheless met the standards of a fair and objective review.
